# Supplementary material for: Poly-glutamine-dependent self-association as a potential mechanism for regulation of androgen receptor activity
Source: PLoS One. 2022 Jan 5;17(1):e0258876. doi: 10.1371/journal.pone.0258876 (PMC8730435; doi:10.1371/journal.pone.0258876)
Supplement: S1 Fig — A. Amino acid sequence of the human androgen receptor N-terminal domain. The poly-Q is highlighted in yellow. B, C. Intrinsically disordered scores predicted for human AR (B) and human KDM4A (C) by the disordered prediction tool IUPred3 [Erdos G, Pajkos M, Dosztanyi Z. IUPred3: prediction of protein disorder enhanced with unambiguous experimental annotation and visualization of evolutionary conservation. Nucleic Acids Res. 2021;49(W1):W297-W303]. (PDF) [file pone.0258876.s001.pdf]

A

1-MEVQLGLGRV YPRPPSKTYR GAFQNLFQSV REVIQNPGPR HPEAASAAPP
GASLLLLQQQ QQQQQQQQQQ QQQQQQQQQQ ETSPRQQQQQ QGEDGSPQAH
RRGPTGYLVL DEEQQPSQPQ SALECHPERG CVPEPGAAVA ASKGLPQQLP
APPDEDDSAA PSTLSLLGPT FPGLSSCSAD LKDILSEAST MQLLQQQQQE
AVSEGSSSGR AREASGAPTS SKDNYLGGTS TISDNAKELC KAVSVSMGLG
VEALEHLSPG EQLRGDCMYA PLLGVPPAVR PTPCAPLAEC KGSLLDDSAG
KSTEDTAEYS PFKGGYTKGL EGESLGCSGS AAAGSSGTLE LPSTLSLYKS
GALDEAAAYQ SRDYYNFPLA LAGPPPPPPP PHPHARIKLE NPLDYGSAWA
AAAAQCRYGD LASLHGAGAA GPGSGSPSAA ASSSWHTLFT AEEGQLYGPC
GGGGGGGGGG GGGGGGGGGG GGGEAGAVAP YGYTRPPQGL AGQESDFTAP
DVWYPGGMVS RVPYPSPTCV KSEMGPWMDS YSGPYGDMRL ETARDHVLPI
DYYFPPQKT**-559**

B

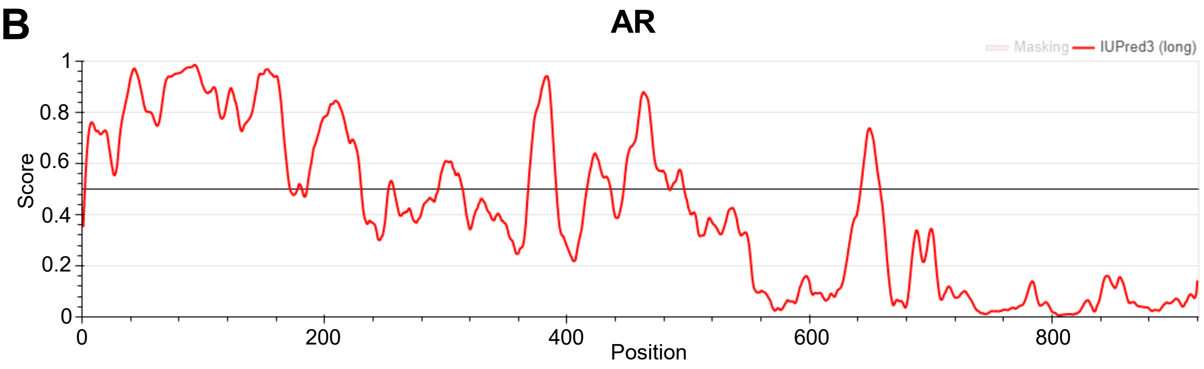

C

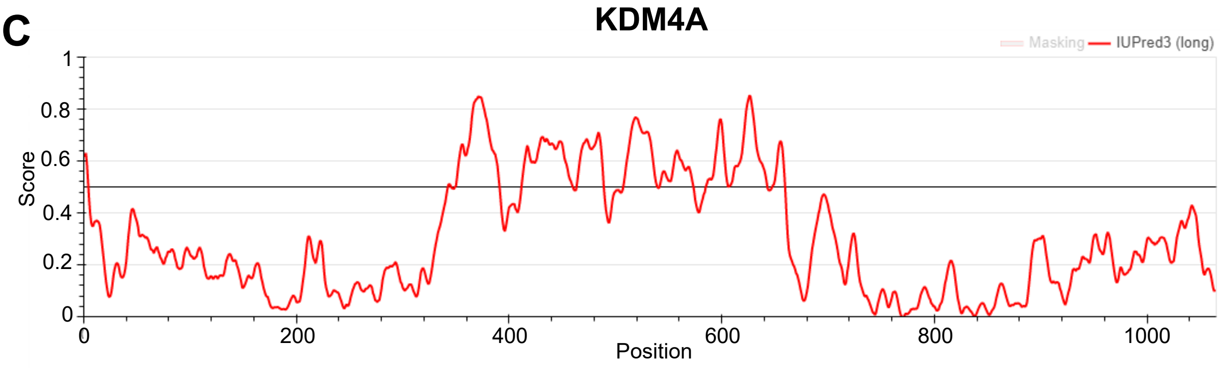

S1 Fig
Roggero et al.
