# Supplementary figures and images for: Poly-glutamine-dependent self-association as a potential mechanism for regulation of androgen receptor activity
Source: PLoS One. 2022 Jan 5;17(1):e0258876. doi: 10.1371/journal.pone.0258876 (PMC8730435; doi:10.1371/journal.pone.0258876)

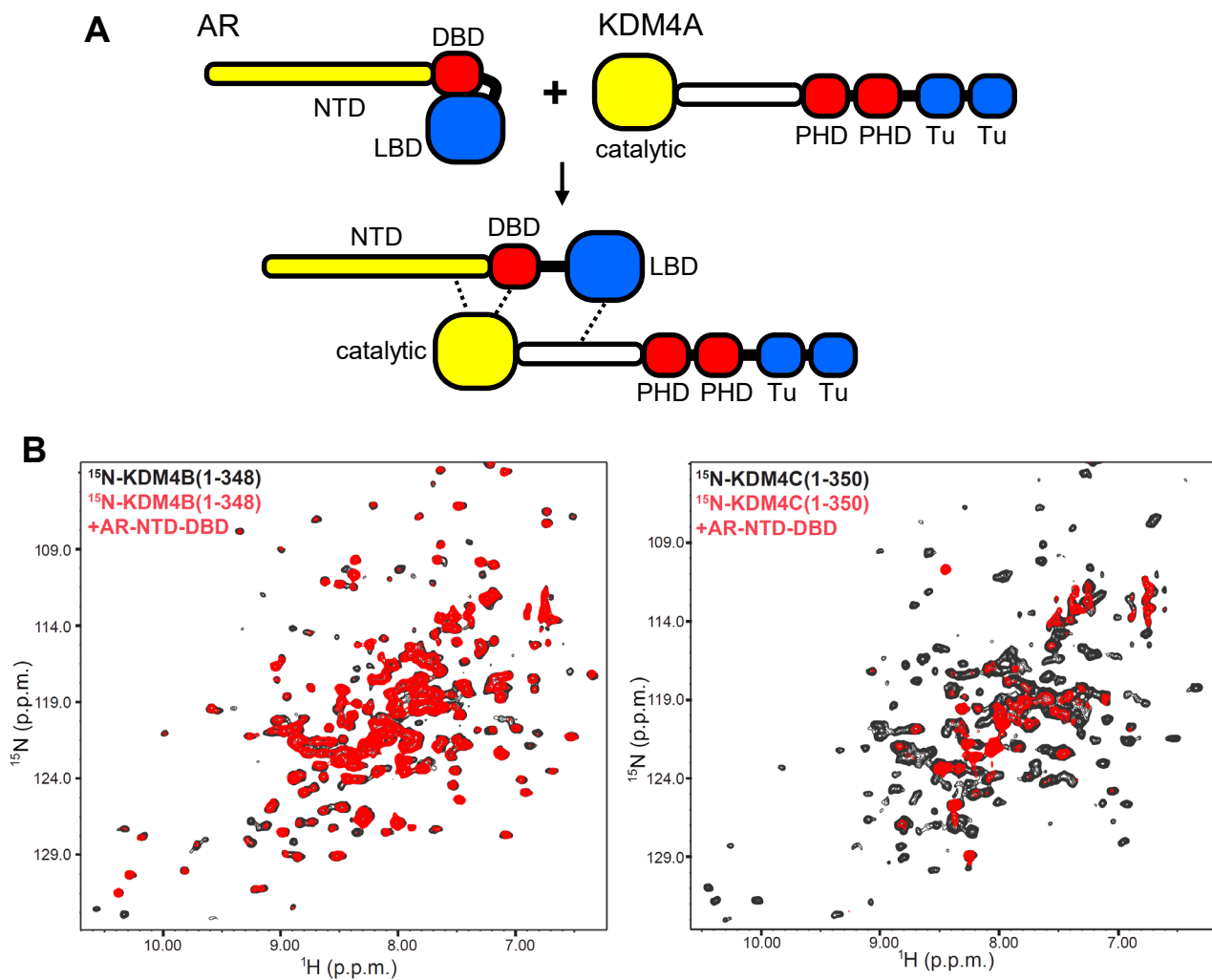

**S2 Fig**  
**Roggero et al.**

Supplement: S2 Fig — A. Model of how AR interacts with KDM4A. The model predicts that binding involves interactions of the AR NTD and DBD domains with the catalytic domain of KDM4A, as well as of the AR LBD with the region of KDM4A spanning residues 301–708. Intramolecular interactions between the DBD and LBD domains are postulated to hinder the DBD/KD4MA interactions. B. The diagrams show superpositions of 1H-15N TROSY-HSQC spectra of 15N-KDM4B(1–348) (left) or 15N-KDM4C(1–350) (right) alone (black contours) or in the presence of equimolar amounts of unlabeled AR-NTD-DBD (red). (PDF) [file pone.0258876.s002.pdf]

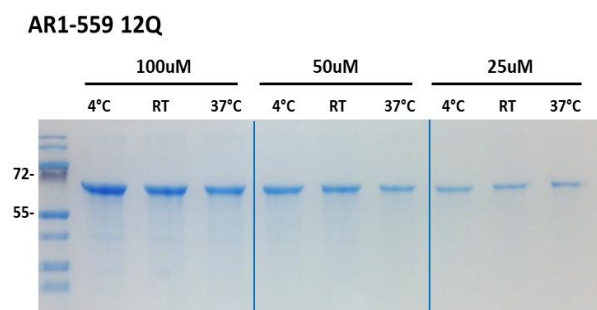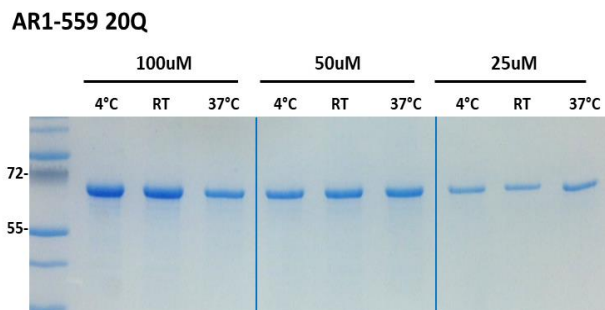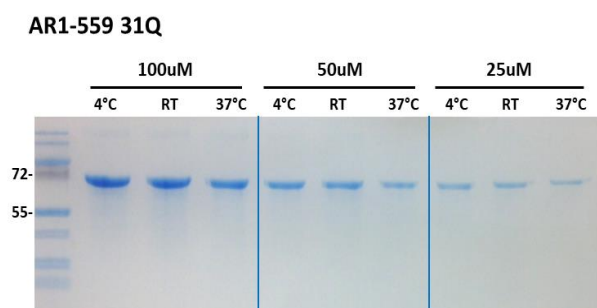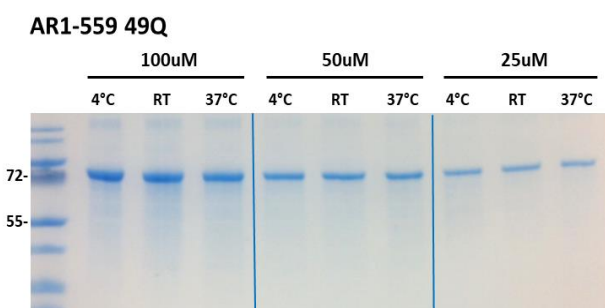

**S3 Fig**  
**Roggero et al.**

Supplement: S3 Fig — The samples were separated by SDS PAGE followed by coomassie blue staining. Molecular weight markers are on the left of each gel. (PDF) [file pone.0258876.s003.pdf]

Figure 3B

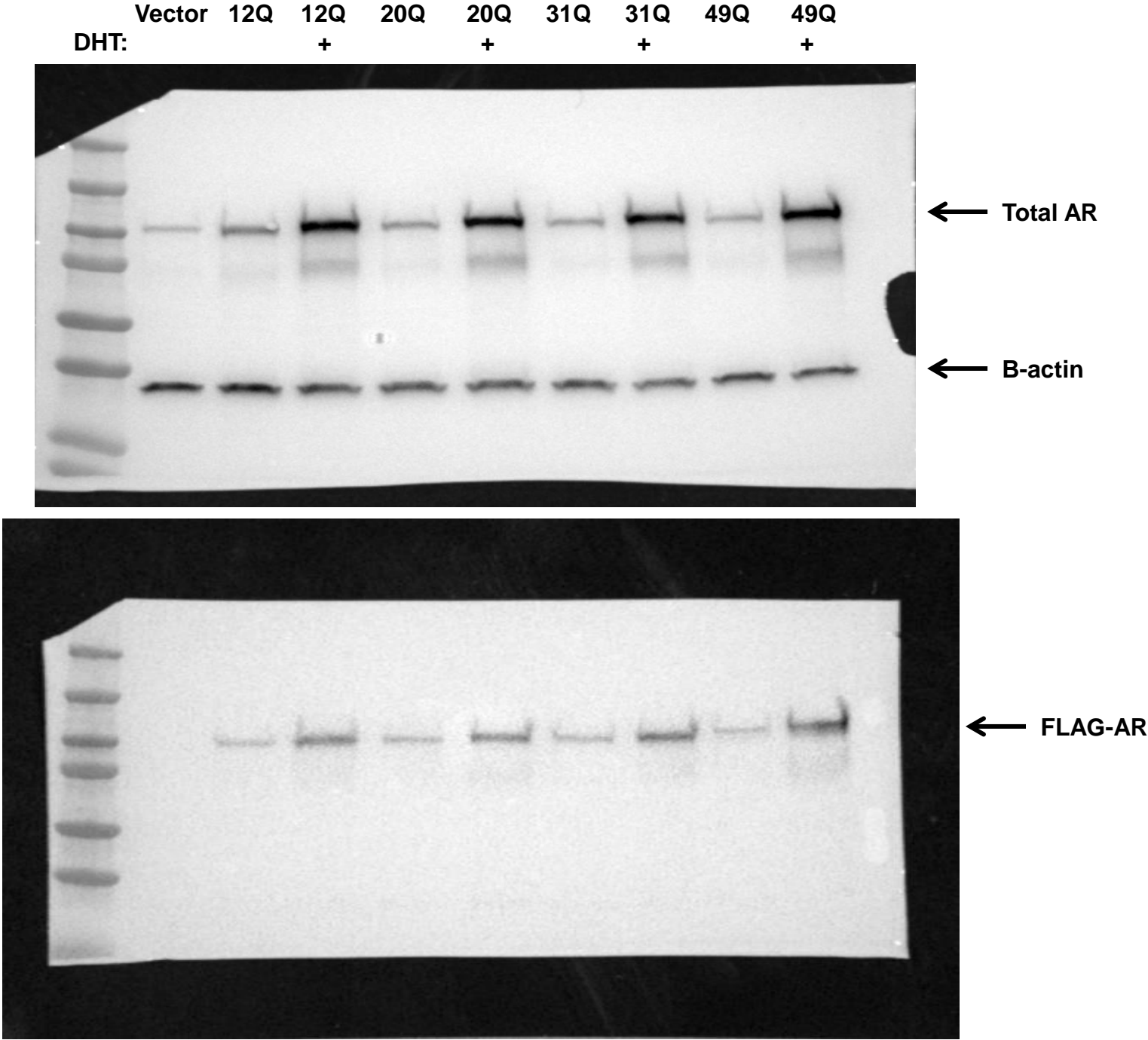

Figure 4C

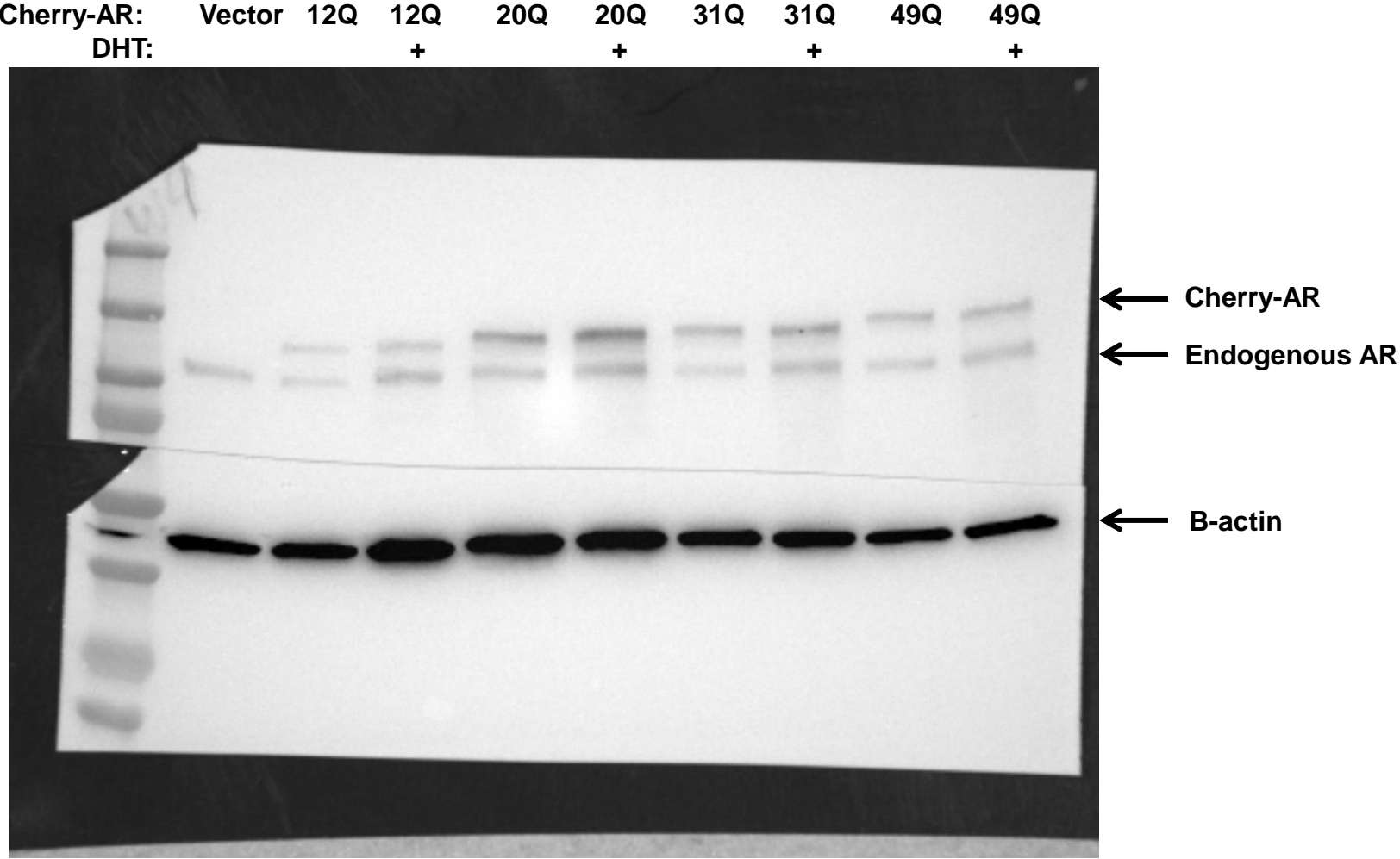

### S3 Fig

AR1-559 12Q

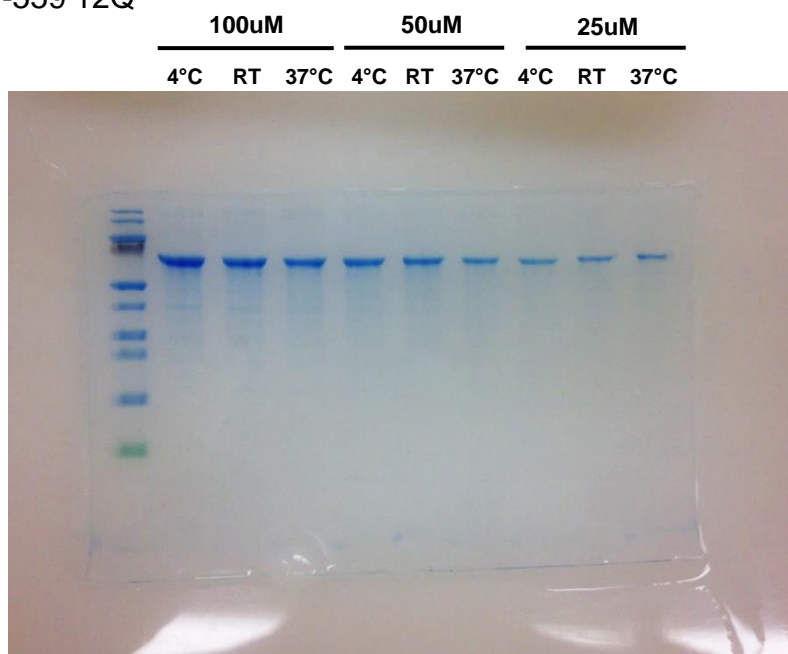

AR1-559 20Q

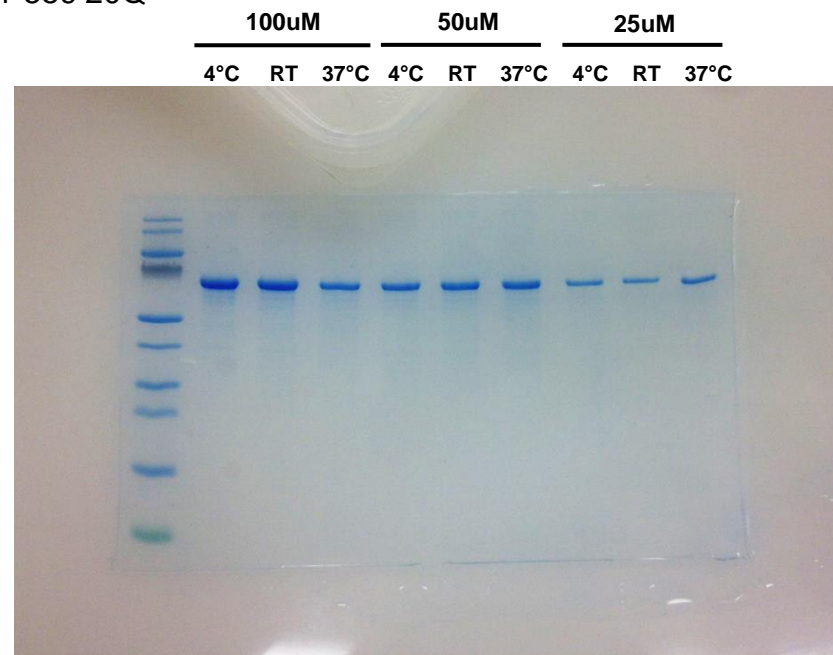

AR1-559 31Q

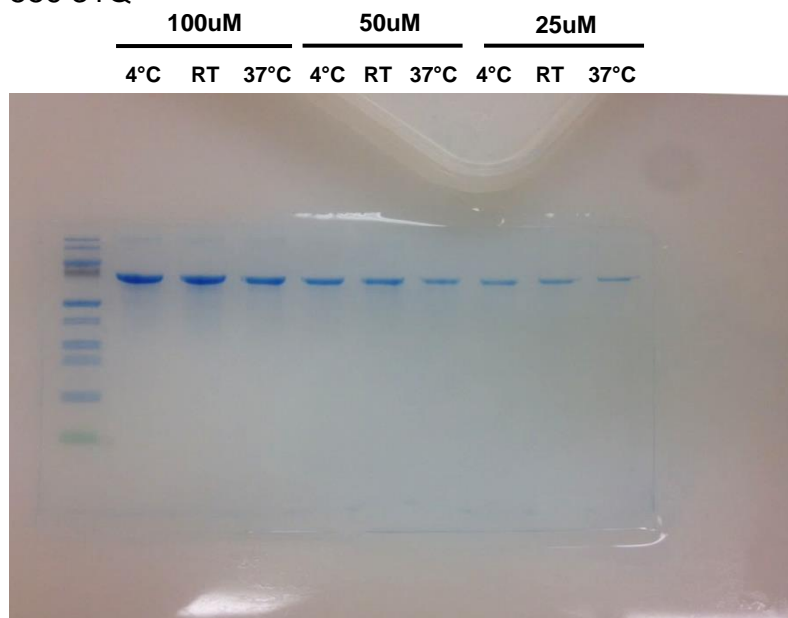

AR1-559 49Q

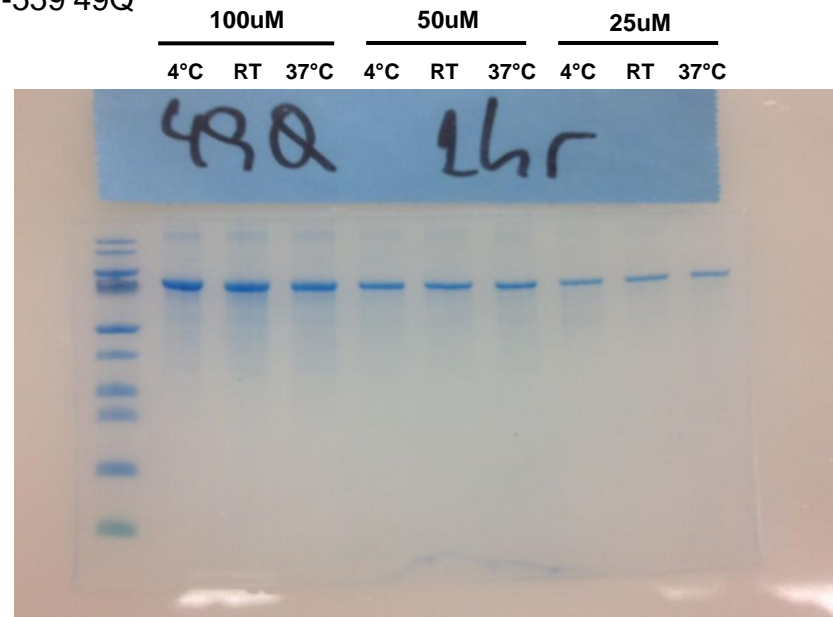

Supplement: S1 Raw images — (PDF) [file pone.0258876.s004.pdf]
