# Supplementary material for: Poly-glutamine-dependent self-association as a potential mechanism for regulation of androgen receptor activity
Source: PLoS One. 2022 Jan 5;17(1):e0258876. doi: 10.1371/journal.pone.0258876 (PMC8730435; doi:10.1371/journal.pone.0258876)
Supplement: Attachment — Submitted filename: review_Rizo_PLOS_2021.pdf [file pone.0258876.s005.pdf]

In their manuscript entitled, “Poly-glutamine-dependent self-association as a potential mechanism for regulation of androgen receptor activity,” Rizo et al investigate the implications of various forms of androgen receptor on interactions with an epigenetic enzyme, transcriptional output, aggregation, and cellular localization. This is of importance as these types of changes alter prostate cancer progression but are not well understood. Specifically, the authors carried out the following experiments: a form of TROSY NMR to determine which domains and polyq NTD lengths affected binding with KDM4 (and isoforms), cross-linking of these as confirmation of the NMR, probed phase separation in vitro as a consequence of NTD AR polyq alteration, phase separation due to increasing poly q length was shown to be reversible (so as to be different from amyloid formation), transcriptional alteration (by means of transactivation assays) were used to show how polyq extensions altered transcription, and different polyq lengths on NTD of AR were monitored in the cell to show the degree of nuclear localization and the formation of punctate structures in the nucleus with varying degrees of polyq sequence. The experiments are of high quality and the scientific conclusions are well justified. It is also important to note that these experiments, due to the difficult nature of working with, purifying, and handling AR are not trivial and require much work. The paper is well written and easy to follow with very few errors. I recommend publication with just some very small changes:

-The word dramatic in the abstract could probably be changed to something more precise such as extensive

-In the abstract it might be good to define low-complexity sequences and why only polyQ is the one to look at here

-In the abstract, KDM4 is not introduced. It should be given a small introduction/rationale for study

-In the intro, paragraph starting line 62, it might be useful for the reader to have the term AF (activation function) introduced along with coregulators. Just a sentence or two so that the general reader knowns how and where and which type of coregulators are recruited to incite transcription.

-The term intrinsically disordered is not mentioned here regarding AF1. Is this because it has some structure? More detail on this especially in regard to other NRs (one example that is well studied for example is ER) and the intrinsically disordered nature and differences between receptors could be useful.

-It wasn’t clear what the mechanism of addition of polyq sequences in a cancer setting is? What is known about how poly q sequences are added to AR? A very small section in intro might help clear this up to the reader, even if it is unknown.

-Line 112 define what a Tudor domain is

-In the discussion it would be nice to see more discussion about the relationship, in general (not just with AR) about the relationship between in vitro phase separation and cellular effects. Are there references to this that could be explained? There may not be much out there on this subject.

-Page 12, line 259, the comma before however should probably be changed to a semicolon
